# The nightmare of scratch tickets: a case report on Gambling Disorder

**DOI:** 10.1192/j.eurpsy.2025.972

**Published:** 2025-08-26

**Authors:** F. Jarmela de Pina, N. Monteiro, I. Faria, F. Sola, C. Silva

**Affiliations:** 1 Centro de Responsabilidade Integrada de Psiquiatria, Centro Hospitalar e Universitário de Coimbra; 2 Faculdade de Medicina da Universidade de Coimbra, Coimbra, Portugal

## Abstract

**Introduction:**

Gambling disorder (GD) can be defined as the maintenance of gambling behavior despite its negative impact both on the health, social, work and financial aspects of one’s life, with growing importance over other interests, loss of control over this behavior and an intense need of maintaining it. In the last decades, the prevalence of GD has been increasing, while it remains an underdiagnosed and undertreated disorder. Regarding scratch tickets (ST), Portugal is the European country with the biggest per capita spending, more than doubling the European average, with 150€ spent annually.

**Objectives:**

We report the case of a woman with GD and aim to briefly discuss the most recent evidence on this subject.

**Methods:**

Description of clinical case and brief review of the literature on the subject.

**Results:**

G. is a caucasian 66-year-old woman without past psychiatric history. Her first contact with ST happened in 2016, when she started working at a kiosk and spent around 1€/day. In 2017, after going through a divorce, her gambling habits worsened, spending around 70€/day. She spent most of her income on gambling and started developing depressive symptoms. By the end of 2020, she is put in a medical leave of absence and starts seeing a therapist. To better control her spendings, her daughter is appointed as her bank account holder, but the patient relapses a week after returning to work, having contracted a loan to keep up with her addiction. She maintains this behavior until 2023 when she is referenced to a Psychiatry appointment and starts treatment, including individual psychotherapy sessions and weekly Gambling Anonymous meetings. In January 2024, G. joins an in-hospital patient group for gambling addicts and decides to retire, to distance herself from the environment that potentiates her addiction. She is now in remission and has been abstinent from gambling for over a year. She still has occasional thoughts about gambling as well as craving to gamble, but all other psychiatric symptoms have resolved, and she has been discharged from Psychiatry appointments.

**Conclusions:**

The average ST gambler is middle aged, plays weekly and earns less than minimum wage, with the chance of gambling decreasing drastically if monthly wage is >1500€. Additionally, service workers are 55% more likely to gamble ST when compared to their counterparts. With the lack of regulation, growth in publicity and the possibility of anonymity, the prevalence of GD is growing, becoming essential that we detect this disorder promptly so that a multidisciplinary treatment can be implemented.

**Disclosure of Interest:**

None Declared

